# Resolution of Fundic Gland Polyposis following Laparoscopic Magnetic Sphincter Augmentation and Subsequent Cessation of Proton Pump Inhibitors

**DOI:** 10.1155/2015/576263

**Published:** 2015-10-27

**Authors:** Joel R. Brockmeyer, Erin E. Connolly, Richard J. Wittchow, Shanu N. Kothari

**Affiliations:** ^1^Minimally Invasive Bariatric Surgery and Advanced Laparoscopy Fellowship, Gundersen Medical Foundation, 1900 South Avenue, La Crosse, WI 54601, USA; ^2^Department of General and Vascular Surgery, Gundersen Health System, 1900 South Avenue, La Crosse, WI 54601, USA; ^3^Department of Pathology, Gundersen Health System, 1900 South Avenue, La Crosse, WI 54601, USA

## Abstract

Gastric polyps occur from a variety of sources and are found commonly on upper endoscopy. We present the case of a 49-year-old female who presented for evaluation for antireflux surgery with a history of fundic gland polyposis who required twice-daily proton pump inhibitors (PPIs) for control of her gastric reflux. After verifying that she met criteria for surgery, she underwent an uncomplicated laparoscopic magnetic sphincter augmentation placement. With the cessation of PPIs following surgery, the fundic gland polyposis resolved. Fundic gland polyps may occur sporadically or within certain syndromes, such as familial adenomatous polyposis. Multiple possible inciting factors exist, including the use of PPIs. This is the first reported case of the resolution of numerous fundic gland polyps following the completion of laparoscopic magnetic sphincter augmentation.

## 1. Introduction

Gastric polyps occur from a variety of sources and are found commonly on upper endoscopy. Due to the high prevalence of gastroesophageal reflux disease in the United States, many patients receive proton pump inhibitors (PPIs). A possible side effect of PPIs is the development of fundic gland polyps [1]. We describe a patient with multiple fundic gland polyps who had resolution of the polyps with the use of magnetic sphincter augmentation leading to cessation of PPIs.

## 2. Case Presentation

**Figure 1:**
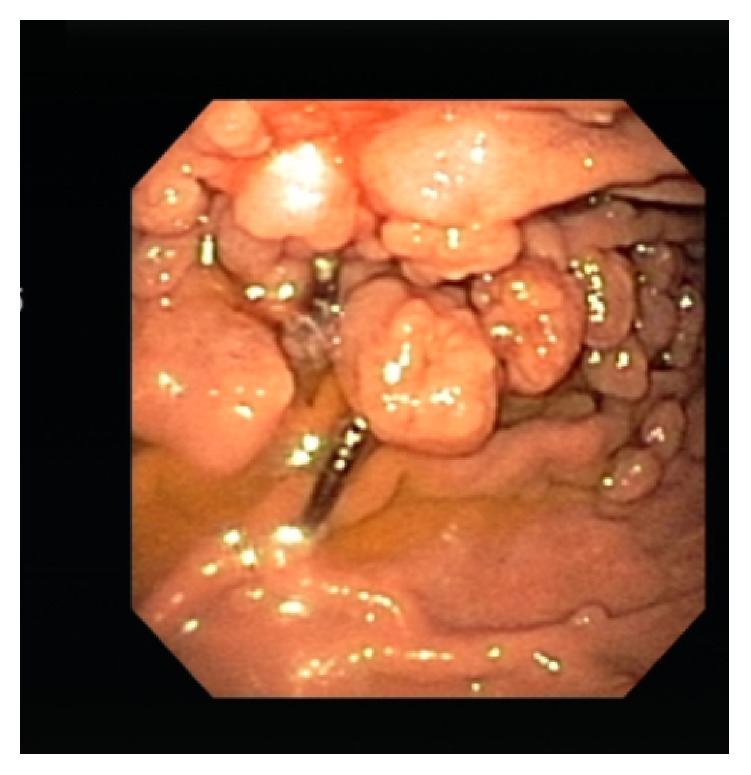
Upper endoscopy image taken prior to magnetic sphincter augmentation that shows multiple large polyps. Biopsy showed fundic gland polyps.

A 49-year-old female presented for evaluation for antireflux surgery. She had a 20-year history of heartburn and frequent acid brash. Until recently, her symptoms were well controlled with twice-daily PPI use with occasional additional over-the-counter antacids used. She had undergone upper endoscopy multiple times over the past 20 years that revealed short segment Barrett esophagus and numerous polyps ranging in size from ten to twenty millimeters (Figure 1).

**Figure 2:**
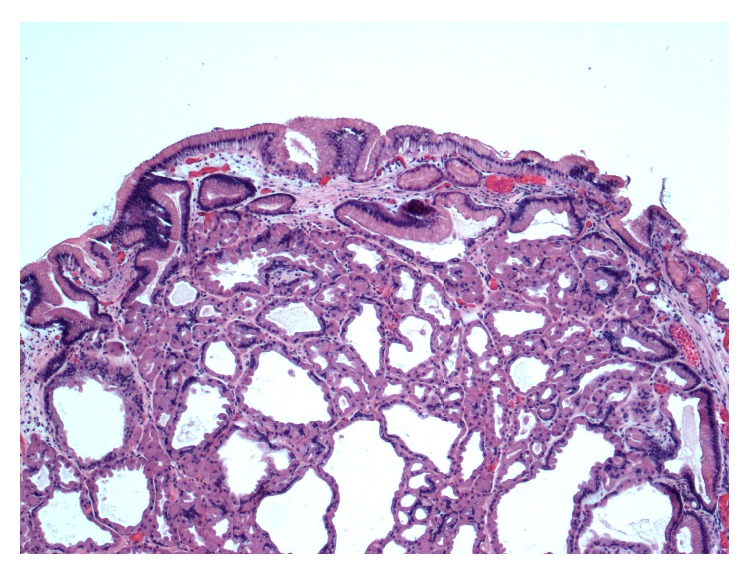
Fundic gland polyp, Hematoxylin and Eosin stain, 40x magnification.

Previous endoscopic biopsies of the innumerable stomach polyps showed fundic gland polyps. She was followed up by gastrointestinal medicine, with upper endoscopy and biopsies yearly. The most recent upper endoscopy one month before surgical evaluation revealed a normal appearing esophagus and innumerable polyps between 10 and 20 mm in size within the body of the stomach. Five of the largest gastric polyps were resected. Pathologic evaluation showed benign fundic gland polyps (Figure 2).

She underwent an esophageal impedance study after stopping her PPI for three weeks. Her DeMeester score was calculated as 20.6 (normal less than 14.7). Acid exposure time was increased in the upright position, 11.7% (normal less than 6.3%). Her total acid exposure time was elevated to 6.8% (normal less than 4.2%). The patient had a positive reflux symptom index for stomach burning of 100% and a positive reflux symptom association probability for heartburn of 100%. Esophageal manometry showed normal esophageal motility. Fluoroscopic upper gastrointestinal swallow study showed reflux without any hiatal hernia or other anatomic defects.

After discussion with the patient regarding laparoscopic fundoplication versus laparoscopic magnetic sphincter augmentation (MSA) (LINX, Torax Inc., Minneapolis, Minnesota, USA), the patient underwent laparoscopic MSA. The operation was completed without incident, and a 15-bead MSA device was placed around the lower esophageal sphincter. She was instructed to eat frequently to actuate the device at least every 4 hours. All antacid therapy was discontinued. She was seen in follow-up three weeks after surgery where she reported being free of reflux symptoms with only occasional dysphagia about three times per week.

**Figure 3:**
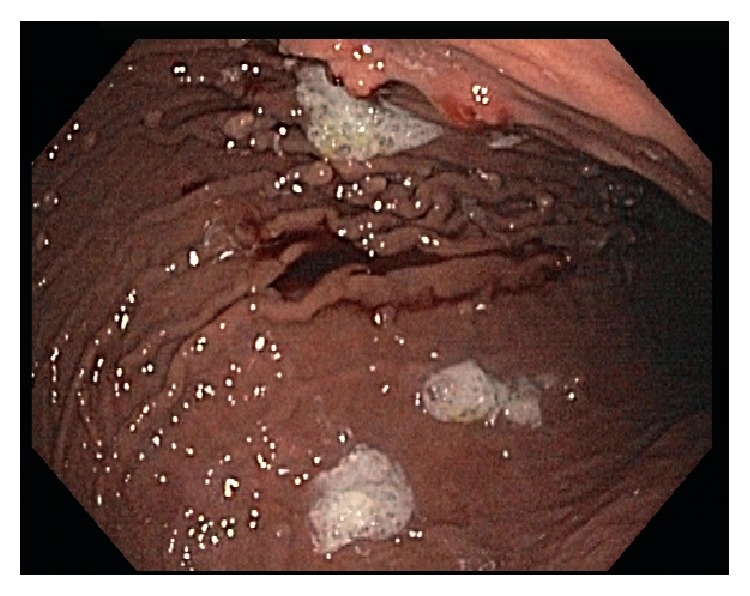
Upper endoscopy image following magnetic sphincter augmentation and cessation of all antacid medications showing minimal polyps that were biopsied and showed hyperplastic polyps.

**Figure 4:**
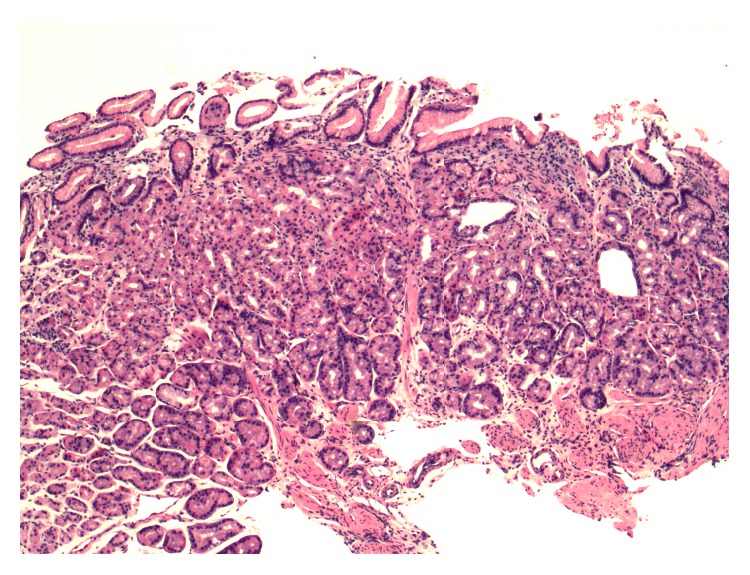
Mild chronic gastritis and reduction of fundic gland polyps, Hematoxylin and Eosin stain, 40x magnification.

The patient was seen nine months after MSA in the gastroenterology clinic for surveillance of her gastric polyps. She remained off all of her antacid medications and had no recurrent symptoms during that time. Surveillance upper endoscopy revealed a grossly normal esophagus. Biopsies of the gastroesophageal junction were obtained, revealing only benign mucosa with no further Barrett epithelium identified. Multiple polyps less than 5 mm in size were found in the body of the stomach with a drastic reduction in the number of polyps found prior to her MSA (Figure 3). Biopsies of these polyps revealed only mild chronic gastritis and a reduction of fundic gland polyps (Figure 4). The remainder of the endoscopy was normal. She has continued to do well since surgery without return of her reflux symptoms.

## 3. Discussion

Fundic gland polyps are the most common gastric polyps and account for about half of all benign gastric polyps [2]. First described in 1977, they are usually located in the body or fundus of the stomach and appear as sessile, shiny, translucent, pale to pinkish in color polyps with tiny surface blood vessels [3]. The polyps can be single or multiple and are characterized by cystically dilated fundic glands lined by flattened parietal cells, chief cells, and mucous neck cells [2]. No dysplastic morphology is seen in the overlying surface and foveolar gastric epithelium. Inflammatory changes in the polyps are minimal or absent and the surrounding gastric tissue is usually normal [3].

Fundic gland polyps may be sporadic or syndromic and are associated with familial adenomatous polyposis (FAP) and attenuated familial adenomatous polyposis (AFAP) [4]. Sporadic polyps are found on about 2% of all upper endoscopies and in 12.5 to 84% of patients with FAP [2]. In FAP-associated fundic gland polyps, inactivating mutations in the APC tumor suppressor gene on chromosome 5q have been found. In sporadic fundic gland polyps, activating mutations of the *β*-catenin gene are sometimes found. The difference in the genetic etiology of fundic gland polyps likely causes the difference in the dysplasia risk between the two, as FAP- and AFAP-associated fundic gland polyps have been found to have 41% rate of dysplasia. Sporadic fundic gland polyps have minimal risk of malignant transformation and rarely any dysplasia [4].

Few factors have been found to correlate with the development of sporadic fundic gland polyps. A negative relationship between* H. pylori* infection and fundic gland polyps has been found [4]. No definite predilection has been found based on sex or race [3]. The association with PPIs has been postulated, first by Dr. Graham in 1992 [5]. This has been substantiated by further studies. Jalving and colleagues performed a prospective study on 599 consecutive patients undergoing upper endoscopy [6]. They found that long-term PPI use was associated with an increased risk of fundic gland polyps. Patients on PPIs for 1–5 years had an odds ratio (OR) of 2.2 (95% CI: 1.3–3.8) of having fundic gland polyps on upper endoscopy, while patients on PPIs for longer than 5 years had an OR of 3.8 (95% CI: 2.2–6.7). Short-term therapy was not associated with development of fundic gland polyps. A similar conclusion was made by Ally et al., in a retrospective study of elective upper endoscopy results. They found a risk of developing fundic gland polyps when patients had been on PPIs for two years or longer [1]. Zelter and colleagues performed a large prospective study on 1780 patients who underwent upper endoscopy over a one-year period [7]. They found gastric polyps in 129 patients (7.2%) with 77 of those polyps identified as fundic gland polyps. Proton pump inhibitor use was detected in 49 patients with fundic gland polyps (63.6%) and 264 patients without fundic gland polyps (15.5%). In their study, women and older patients (58 years versus 50 years) were more likely to develop fundic gland polyps. Proton pump inhibitor use was the most significant predictor for development of fundic gland polyps. A possible mechanism for the development of fundic gland polyps in patients on PPIs was proposed by Cats et al., who suggested that fundic gland polyps develop as a consequence of mechanical obstruction of gastric glands by hyperplastic parietal cells [8]. Long-term administration of PPIs has been shown to cause parietal cell hyperplasia secondary to PPI-induced hypergastrinemia [9]. Protrusion of hyperplastic parietal cells into the glandular lumina of gastric fundic glands causes narrowing and obstruction of the glandular lumina and cystic dilatation of fundic glands, characteristic of fundic gland polyps [8].

Despite the proposed mechanism, some debate still exists about the causation of fundic gland polyps by long-term PPI use. Vieth and Stolte completed a retrospective study over a one-year period [10]. The frequency of fundic gland polyps in 2,251 patients on PPI therapy without* H. pylori* infection was compared to a control group of 28,096 patients not on PPI therapy without* H. pylori* infection. The rate of fundic gland polyps was the same in the two groups, 5.2% in PPI group and 5.0% in control group. No other significant differences were found between the two groups in relation to gastritis, age, or sex. Bearing this study in mind, more studies seem to show a correlation between long-term PPI use and fundic gland polyp development than not.

In fundic gland polyps associated with FAP, predilection for dysplasia and development of gastric adenocarcinoma is known to exist [1]. While the vast majority of sporadic fundic gland polyps are benign and pose no risk of malignant degeneration, several cases of malignant degeneration have been reported in the literature. Low-grade and high-grade dysplasia have been found within fundic gland polyps in large series [11, 12]. Those cancers that do develop in fundic gland polyps may be a different subtype of gastric adenocarcinoma [13]. Jeong and colleagues also describe a case of a fundic gland polyp containing signet-ring cell carcinoma. While these rare cases have been reported of cancer arising within a fundic gland polyp, these polyps are still considered benign.

By comparison, hyperplastic polyps (HPPs) are a very common gastric polyp and the most common epithelial polyp of the stomach [14]. They are found throughout the stomach and are sometimes mistaken endoscopically for cancer [15]. Hyperplastic polyps have a slight preference for the antrum [14]. Hyperplastic polyps arise in mucosa affected by chronic inflammation, such as autoimmune gastritis,* H. pylori* gastritis, or postantrectomy stomach [15]. Microscopically, HPPs contain hyperplastic, elongated, or dilated foveolae that are mixed with variable amounts of inflamed stroma. The foveolae lining is composed of mature gastric mucus cells with abundant cytoplasm, except in areas of surface erosions, where nuclear enlargement and depletion of cytoplasmic mucin may cause a regenerative appearance [16]. The genetic pathogenesis of HPPs remains largely unknown with the K-*ras* protooncogene a potential source. The suggested pathogenesis is that HPPs are the result of prominent, reparative, or regenerative phenomena [14]. The rate of dysplasia or adenocarcinoma within HPPs is relatively rare but has been reported in the literature at 1.9%–19% [15]. Most studies seem to suggest that larger HPPs are more likely to harbor adenocarcinoma [14].

Magnetic sphincter augmentation is a newer technology for the surgical treatment of reflux disease in patients with normal anatomy and absence of large hiatal hernia. It has been shown to be a safe procedure with minimal complications, the most common being dysphagia [17]. Patients are candidates for the surgery if they have typical gastroesophageal reflux disease symptoms that are documented on esophageal pH studies and normal esophageal manometry studies and a hiatal hernia less than 3 cm in size and use a daily PPI with some relief. Pertinent to this case, patients have had great success at stopping all antacid therapy. Ganz et al. showed a PPI use rate of only 13% at 3 years in the initial MSA study [18].

In this patient, implantation of MSA allowed for cessation of her PPI therapy. With the likely correlation between PPI use and the development of fundic gland polyps, the innumerable fundic gland polyps resolved leaving only a few FGPs and mild gastritis that can be more easily followed up.

This is the first reported case of the resolution of numerous fundic gland polyps following the completion of laparoscopic MSA. Due to the successful cessation of proton pump inhibitors in patients following MSA, it should be considered in all eligible patients with multiple fundic gland polyps to allow patients to stop PPIs and have resolution of their reflux symptoms.
